# Resting Systemic Irisin Concentrations Are Lower in Older versus Younger Males after 12 Weeks of Resistance-Exercise Training While Apelin and IL-15 Concentrations Were Increased in the Whole Cohort

**DOI:** 10.3390/muscles3030018

**Published:** 2024-07-01

**Authors:** Dean M. Cordingley, Judy E. Anderson, Stephen M. Cornish

**Affiliations:** 1 Applied Health Sciences, University of Manitoba, Winnipeg, MB R3T 2N2, Canada; umcordid@myumanitoba.ca; 2 Pan Am Clinic Foundation, 75 Poseidon Bay, Winnipeg, MB R3M 3E4, Canada; 3 Faculty of Science, University of Manitoba, Winnipeg, MB R3T 2N2, Canada; judith.anderson@umanitoba.ca; 4 Faculty of Kinesiology and Recreation Management, University of Manitoba, Winnipeg, MB R3T 2N2, Canada; 5 Centre for Aging, University of Manitoba, Winnipeg, MB R3T 2N2, Canada

**Keywords:** inflammation, resistance-exercise, aging, apelin, FGF-21, IL-4, IL-6, IL-7, IL-15, irisin, LIF

## Abstract

Myokines released by exercise are identified as factors that can influence a person’s health and wellbeing. While myokine secretion in response to an acute bout of endurance and resistance-type exercise has been examined, the influence of resistance-exercise training on myokines at rest is less well established. Therefore, this study was designed to evaluate a panel of myokines at rest following a 12-week resistance-exercise training program in younger and older males. Participants (n = 15) completed a 12-week progressive resistance-exercise training program supervised by a certified fitness professional. The training protocol targeted all major muscle groups of the upper and lower body. Resting blood samples were collected before and after completion of the training program to determine concentrations of apelin, fibroblast growth factor-21 (FGF-21), interleukin (IL)-4, IL-6, IL-7, IL-15, leukemia inhibitory factor (LIF), and irisin. Two-way repeated ANOVAs were used to compare variables between time-points and age groups. There was a main effect of time found for apelin (*p* = 0.003) and IL-15 (*p* < 0.001), while no main effects for group or time were found for the other myokines (all *p* > 0.05). Age group × training status interactions were found for IL-6 (*p* = 0.04) and irisin (*p* = 0.014) without pairwise differences for IL-6 (*p* > 0.05), and younger males had higher concentrations of irisin compared to older males post-training (*p* = 0.036). Overall, the 12-week resistance-exercise training program significantly increased apelin and IL-15 over time but did not change the other resting myokine concentrations for the younger or older males. However, the higher concentration of irisin in younger versus older males post-training suggests a potential explanation for the anabolic resistance observed with aging.

## 1. Introduction

Aging is associated with a chronic low-grade inflammatory state which may have a negative influence on skeletal muscle by promoting the development of sarcopenia (the loss of skeletal muscle mass and function with age) [1]. While various interventions (nutrition, various nutrients, pharmacological) have been studied to ameliorate sarcopenia in older adults, few have had substantial effects in reducing the development of sarcopenia in this population. Conversely, resistance-exercise training has been shown to be beneficial in improving skeletal muscle function and mass in older adults [2]. Resistance-exercise training provides the stimulus necessary for maintaining and enhancing skeletal muscle mass and function as well as other health benefits to numerous physiological systems [2]. The effect of resistance-exercise training on resting concentrations of various cytokines has been a research topic of interest for the past few years [3,4]. Generally, although simplistically, it is thought that pro-inflammatory cytokines will decrease at rest after resistance-exercise training while anti-inflammatory cytokines may increase (i.e., an anti-inflammatory environment is produced) [5,6]. The effects of various types of exercise training on pro- and anti-inflammatory biomarkers of inflammatory processes continue to be investigated [7,8]. A recent systematic review evaluated the effects of resistance training, aerobic training, or combined training on interleukin-6 (IL-6), tumor necrosis factor-alpha (TNF-α), and C-reactive protein (CRP) in older adults with and without chronic diseases [9]. It was found that only combined training reduced concentrations of IL-6, while aerobic training decreased TNF-α, and all three training methods decreased CRP concentrations [9]. However, there is currently a paucity of literature that utilize a research design to directly compare the influence of resistance-exercise and aerobic-exercise training on pro- and anti-inflammatory biomarkers in a single study. While many cytokines play a role in the inflammatory process, those released from skeletal muscle tissue (called myokines) are of increasing relevance considering literature on endocrine communications between skeletal muscle and the immune system [10,11].

Many myokines have been associated with forceful skeletal muscle contraction, such as during resistance exercise [4,12]. IL-6, IL-15, and irisin have been extensively researched [5,7,8,10,12,13]; however, others, such as apelin, FGF-21, IL-4, IL-7, and leukemia inhibitory factor (LIF), have not been investigated as fully. Most myokines are considered pleiotropic in nature and IL-6 is no exception as it has multiple physiological roles including potentially pro-inflammatory and anti-inflammatory functions [14] and has effects on skeletal muscle growth and hypertrophy [12]. Irisin is known for its effects on the ‘browning’ of white adipose tissue [12] but may also enhance anti-inflammatory activity in metabolic syndrome [8]. Further, IL-4 is an anti-inflammatory myokine and appears to have an anabolic role in skeletal muscle [15]. IL-7 is considered essential for T-cell, B-cell, and natural killer cell (NK-cell) growth within the immune system; it has pro-inflammatory effects and may also play a key role in skeletal muscle satellite-cell activity [16,17,18]. IL-15 is theorized to be both anabolic and anti-atrophic in muscle [13]. Apelin seems to be involved in ameliorating sarcopenia by enhancing satellite cell function, at least in an aging rodent model [19], and producing an anti-inflammatory effect [20]. Moreover, the myokine, LIF, a member of the IL-6 family [21], participates in skeletal muscle growth [22] and is pro-inflammatory by stimulating acute phase proteins [23]. Furthermore, a rodent model demonstrated FGF-21 to be anti-inflammatory and the deficiency of this myokine led to skeletal muscle atrophy [24].

Despite the potent physiological effects of the above-mentioned myokines, we do not know (or it is not understood) how resting/basal concentrations of such myokines may be affected by resistance-exercise training especially in aging. The objective of this study was to evaluate a panel of myokines (apelin, FGF-21, IL-4, IL-6, IL-7, IL-15, irisin, and LIF) to determine if 12 weeks of progressive, whole-body resistance-exercise training has any substantial effect on their resting concentrations, and to compare responses in younger and older males. We hypothesized that older males but not younger males would show a decrease in pro-inflammatory myokines (IL-6, IL-7, LIF) and an increase in anti-inflammatory myokines (apelin, FGF-21, IL-4, IL-15, irisin) after completing 12 weeks of a standardized resistance-exercise training program.

## 2. Results

**Figure 1:**
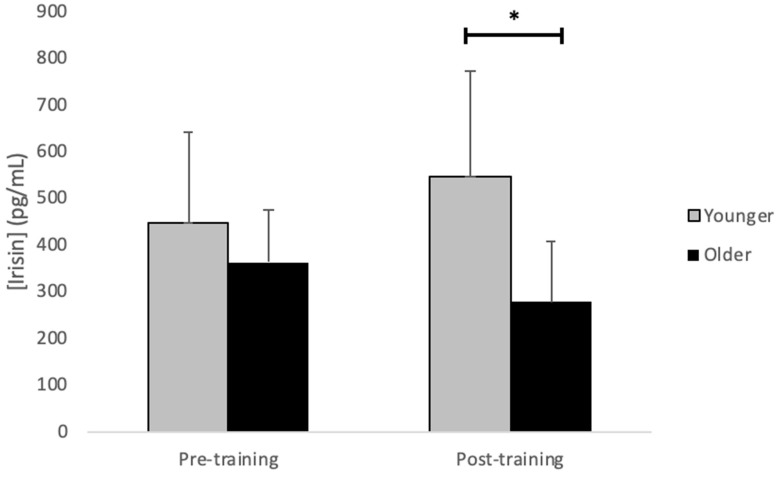
Significant group × training status interaction (*p* = 0.014) for irisin. * Indicates younger males having greater irisin concentrations post-training than older males.

The mean age of the younger and older groups were 24.9 ± 3.9 years and 68.3 ± 5.0 years, respectively. At baseline, there were no differences in any anthropometric measures (younger: height = 179.6 ± 11.7 cm, body mass = 90.89 ± 20.04 kg, body mass index = 28.1 ± 4.8 kg/m^2^, body fat percentage = 21.1 ± 8.9%; older: height = 177.2 ± 3.3 cm, body mass = 90.53 ± 11.28 kg, body mass index = 28.8 ± 3.6 kg/m^2^, body fat percentage = 25.8 ± 3.8%; all *p* > 0.05). The descriptive statistics for each myokine (mean ± SD) by time (pre- and post-training) and group (younger versus older males) are shown in Table 1. Overall, there were no main or age group effects for the following six myokines (FGF-21, IL-4, IL-6, IL-7, irisin, and LIF; all *p* > 0.05; see Table 1). However, there were main effects of time for apelin (*p* = 0.003) and IL-15 (*p* < 0.001) where both myokines increased from pre- to post-training (*p* = 0.005 and *p* = 0.0006, respectively; see Table 1). Furthermore, for irisin, there was a significant time × age group interaction (*p* = 0.01) with irisin concentrations being lower post-training in older males (n = 7) compared to in younger males (*p* = 0.036, n = 8 see Figure 1). There was also a significant interaction of time × age group for IL-6 (*p* = 0.04), although *post hoc* analysis did not reveal a significant pairwise difference between the two age groups (*p* > 0.05). Notably, the training program increased fat-free mass, average strength, and strength per kg of fat-free mass, and decreased body fat (see previously published study for detailed results [25]).

## 3. Discussion

The main finding of this study was that resting plasma concentrations of apelin and IL-15 were significantly increased over time in our whole cohort following a 12-week resistance-exercise training program while the other six myokines (FGF-21, IL-4, IL-6, IL-7, irisin, and LIF) did not change significantly over time and there were no main effects for age group. This finding partially supports our hypothesis that 12 weeks of resistance-exercise training would increase anti-inflammatory myokines in older males; however, the increase in apelin and IL-15 in the entire cohort (i.e., younger, and older) was unexpected. Interestingly, following a 12-week resistance-exercise training program, older males had lower resting plasma irisin concentrations compared to younger males, which does not support the hypothesis that another anti-inflammatory myokine (irisin) would increase in our older cohort. These findings suggest that although some myokines may respond similarly to resistance training in younger and older adults, the response may not be uniform across age groups for all myokines.

Mechanistically, the increased concentrations of apelin and IL-15 could have beneficial impacts on skeletal muscle. Apelin supports myogenesis and angiogenesis [26], increases mitochondrial ATP production and the expression of mitochondrial genes in myotubes [27], and may be required for the activation of the IGF-1 signaling pathway in response to exercise training [27]. In skeletal muscle, IL-15 is anabolic, supports myogenesis and could mitigate skeletal muscle loss due to inflammation [28,29], and can induce skeletal muscle hypertrophy [30]. However, although apelin and IL-15 are both anabolic and support skeletal muscle growth, they work through distinct pathways. As previously noted, apelin supports the activation of the IGF-1 signaling pathway [27], but the actions of IL-15 are distinct from the IGF-1 pathway [30]. This suggests that both apelin and IL-15 could be unique targets for treating age- and disease-related muscle loss.

A particularly interesting outcome from the study is that the mean concentration of irisin at rest differed between younger and older males after but not before the resistance-exercise program. The absence of a change in irisin concentration with exercise training in older males goes against previous research which found a two-fold increase in plasma irisin following 10 weeks of endurance-cycle training [31]. A recent systematic review using meta-analysis reported that resistance training, either alone or combined with endurance-exercise training, increased systemic irisin concentrations [32]. Differences with the current results may be due to the precise type of exercise training employed, since the study by Boström et al. [31] used endurance-type training while the current study used resistance-type training. Current findings are consistent with another report in which serum irisin levels in children did not change after either a 6-week or 1-year physical activity intervention [33].

Additionally, finding that younger and older males had different irisin concentrations following the 12-week training period suggests an interesting facet of age-related physiology. Irisin concentrations in younger and older adults do not respond the same to endurance exercise [34]. However, previous studies have found that endurance exercise results in elevated serum concentrations of irisin in middle-aged and older adults but not younger adults [34], which is opposite to the trend observed in the current study. A decrease in circulating irisin in the older males in our study could indicate that less of this myokine is available in the circulation after a period of training with resistance-exercise in the older population. Previous in vitro research showed that irisin is responsible for increasing insulin-like growth factor-1 (IGF-1), which has a strong anabolic action on skeletal muscle tissue [35]. The consequences of less irisin, and then less IGF-1, in the systemic circulation of older males could account for a lower anabolic activity of this myokine and, thus, less preservation of the metabolic balance of skeletal muscle protein compared to the physiology of the exercise response in younger males. Such a distinction may relate to the anabolic resistance that older adults experience as they age and could partially explain why older adults are less able to preserve skeletal muscle mass and strength [36]. Interestingly, the average fat-free mass improvement in younger males completing our 12-week resistance-exercise training program was ~1.5 kg, whereas for the older males it was ~0.7 kg [25], which does suggest less adaptation occurred in the older male participants.

The finding that a 12-week resistance-exercise training program did produce changes in some, but not all, resting myokine concentrations was unexpected. The absence of change in certain myokines (FGF-21, IL-4, IL-6, IL-7, irisin, LIF) while apelin and IL-15 significantly increased in the entire cohort over time is not consistent with common understanding. Generally, it is thought that chronic exercise training will decrease pro-inflammatory associated myokines and increase myokines associated with an anti-inflammatory state [5,6]. One possible interpretation of these results is that adaptations to chronic resistance-exercise training may be myokine specific. Apelin and IL-15 have both been shown to have pro-myogenic properties [19,37] and our results demonstrate that an increased concentration of these myokines occurs with resistance-exercise training. Hypothetically, the increase in these two myokines could help to create an anti-inflammatory environment but also may be necessary to help promote muscle protein synthesis associated with resistance-exercise training. Previous research investigating IL-15 and its receptor in muscle tissue has found an association between myofibrillar fractional synthetic rate and mRNA of IL-15Rα [37], thus suggesting an influence of one on the other. Another possible explanation for these results is that the participants were initially untrained, and the initiation of the resistance training program may have resulted in a pro-inflammatory response which was later abrogated by a compensatory anti-inflammatory response resulting in no observed change for some myokines. A third possible explanation is that the 12-week progressive resistance-exercise training protocol in the current study was not of an appropriate frequency, intensity, and/or duration to elicit the expected changes in all the myokines. However, the protocol was sufficient to induce improvements in body composition and strength, suggesting it was adequate to positively modify parameters such as myokines thought to play a role in the inflammatory balance during resistance-exercise training.

An alternative explanation for why both apelin and IL-15 concentrations increased from pre- to post-training in the entire cohort may be the influence these myokines have on anabolism in skeletal muscle. Previous research has demonstrated that apelin targets satellite cells during skeletal muscle regeneration in a mouse model and that recombinant apelin supplementation after cardiotoxin injection improved mRNA expression of *Pax7*, *Myf5*, and *Myog* (markers associated with satellite cells) [19]. Further evidence from the previous study revealed that as the percent change in apelin concentration improved from 0 to 6 months with a physical activity intervention in older adults, so did the participants’ short physical performance battery (a test of functional fitness) [19]. The inhibition of apelin’s action results in reduced cross-sectional area of regenerating myofibers compared to a control condition demonstrating that this myokine is necessary for myogenesis during muscle regeneration [26]. Our results suggest that resistance-exercise training can improve apelin concentrations and, thus, may indicate another beneficial effect of exercise in both younger and older males. Interestingly, there was a trend for a time × age group interaction in apelin where the mean increase in apelin was greater in the older versus the younger age group (52% and 14% increase, respectively).

IL-15 also has evidence supporting its role in anabolic activity of skeletal muscle [12,13]. Quinn et al. [38] demonstrated decreases in serum and muscle levels of IL-15 with age in a mouse model, possibly contributing to sarcopenia development. Our study demonstrates that IL-15 in the systemic circulation is increased with 12 weeks of resistance-exercise training in younger and older human males. There was also a trend for a time × age group interaction with IL-15 where the older males increased more than the younger males over the 12-week intervention (67% and 22% average improvements, respectively). This points to the ability of resistance-exercise training to increase the concentrations of some key myokines (i.e., IL-15 and apelin) in older males to concentrations that would be associated with younger males and may be helpful in promoting muscle strength and mass increases and ameliorating sarcopenia. These intriguing, age-specific responses to resistance-exercise training highlight the complexity of predicting the impact of exercise activity on myogenic growth and muscle strength.

The underlying mechanistic reason for the age-related differences in myokine concentrations found in this study were not evaluated but there could be multiple explanations. One possible explanation is that myokine secretion in response to resistance training is associated with muscle volume or muscle quality (i.e., strength per kg muscle mass) where older adults typically have decreased muscle volume and strength. Additionally, the inflammatory state of the skeletal muscle, or other biological systems throughout the body, could possibly be associated with the change in systemic myokine concentrations in response to exercise training. Further research is needed to elucidate the mechanisms underlying age-related differences in myokine adaptations to exercise training.

The current study is not without limitations. First, myokine concentrations were highly variable in the study participants, especially in older males. This was expected as there were likely chronic, low-level inflammatory processes occurring in some of the older males that were yet otherwise healthy. Second, dietary intake was not directly controlled or analyzed. Participants were only requested to consume the same diet throughout the resistance-training period. Diet and nutrient intake can modulate myokine responses to exercise [39,40,41] and, thus, influence study results if participants had varied their diet during their participation in the study. Participants were also asked to consume the exact same diet around the time of their blood draws and to record their dietary intake (3 days) for the baseline blood draw and subsequently asked to repeat that diet for the 3 days before the 12-week blood draw. Nonetheless, it is not known how many of the participants, young or older, may have been unable or unwilling to follow these study instructions. Thus, variations in dietary intake may have limited the present results. Third, beyond diet, other lifestyle factors (such as alcohol consumption, tobacco use, etc.) were not controlled or evaluated and could have influenced participant inflammatory state and the study outcomes. Fourth, the sample size for this study was small and could have influenced the findings. Fifth, the resistance-training program used may have been sufficient to elicit adaptations to certain myokines, but not all. A resistance-training program of a different duration, intensity, and volume may result in a different myokine response than was found in this study. Sixth, the current study only included male participants. This does not allow for the transfer of findings to females. A similar study conducted in females is needed as resistance training results in different adaptations for females and males across the life span [42,43]. Lastly, because blood samples were only collected in a pre-post fashion, it is unknown what changes to myokines may have occurred throughout the 12 weeks of the resistance training program that may have been masked by the time the post-training blood sample was collected.

Overall, while a 12-week progressive resistance-exercise training program increased apelin and IL-15 at rest, it did not modulate the other myokines evaluated in this research. Resting irisin appeared to respond to resistance-exercise training in an age-dependent way. Further investigation of this response is required to evaluate whether combined nutritional or pharmaceutical interventions that accompany resistance-exercise training may enhance the preservation and/or accrual of skeletal muscle mass. The underlying reason for lower resting irisin in older versus younger male adults after 12 weeks of resistance-exercise training remains to be determined. Future research evaluating the effects of exercise training on older adults in relation to the degree of chronic low-grade inflammation in muscle (and elsewhere) could provide new insights into underlying contributions of inflammation responsible for the decline in skeletal muscle mass with age. Additionally, investigation of combined interventions (i.e., combined exercise and nutritional interventions) may provide more opportunities to improve skeletal muscle health with aging. Future studies utilizing a randomized controlled trial study design would benefit this area of research and improve the robustness of conclusions drawn for the current findings.

We conclude that a 12-week resistance-exercise training program is effective at raising the resting concentrations of apelin and IL-15 in the systemic circulation. We further conclude that the concentration of the myokine, irisin, decreases at rest in older but not younger males after participation in a 12-week resistance-exercise training program. Lastly, resting levels of five other myokines (FGF-21, IL-4, IL-6, IL-7, and LIF) were not changed following the resistance-exercise training protocol, but further investigation of these specific myokines would be valuable due to the high variability observed in both younger and older males.

## 4. Materials and Methods

### 4.1. Experimental Design

This study was a secondary analysis of data collected as part of a previously published non-randomized, uncontrolled study from our laboratory [25]. For a detailed study protocol, see the previous report [25]. Study participants had blood drawn prior to and following a 12-week resistance training program. Additionally, participants had serial blood sampling completed following blood-flow-restricted resistance exercise both before and after the 12-week resistance training program, of which the results are reported in our previous publication [25]. This study was approved by the University of Manitoba Institutional Review Board (HS21401) and all participants provided informed consent prior to the initiation of study activities.

### 4.2. Participants

Initially, 18 participants were recruited, with a total of 15 participants completing all study activities. The 3 participants who did not complete all study-related activities were removed from the analysis. Of the participants, 8 were younger males (age range = 18–29 years old) and 7 were older males (age range = 64–76 years old). All participants were: (1) inactive (performed ≤1 structured bout of resistance exercise per week), (2) did not have a previously diagnosed inflammatory disease, (3) were not consuming any anti-inflammatory medications or nutritional supplements containing ingredients with anti-inflammatory properties, (4) free of mental illness and were cognitively able, (5) were physically able to participate in a structured resistance training program, and (6) were free of cardiovascular and peripheral arterial disease.

### 4.3. Training Protocol

The training protocol required participants to perform resistance training exercise 3 days per week for 12 weeks under the supervision of a certified exercise professional. This protocol was used as it has previously been found to induce training adaptations in older males in our lab [44]. The training protocol included both upper (chest press, seated rows, shoulder press, dumbbell bicep curls, and dumbbell overhead triceps press) and lower body (leg press, leg extension, dumbbell squats, and plantar flexion) resistance exercises and was progressive in nature starting with 10 repetitions at 60%, 1 repetition maximum for 2 sets per exercise, and reaching 10 repetitions at 85%, 1 repetition maximum for 3 sets per exercise during the last week [25]. The 1 repetition maximum testing occurred following a 5 min moderate intensity warm-up on a cycle ergometer. Participants began by performing 5–10 repetitions at a weight equivalent to 40–60% of their estimated 1 repetition maximum. A 1 min rest was then provided before the participants performed 3–5 repetitions at a weight that was 60–80% of their estimated 1 repetition maximum. Participants were then provided a 3–5 min rest and then attempted 1 repetition with and increased weight. If successful, participants rested for 3–5 min before attempting another repetition with an increased weight. The participants’ 1 repetition maximum was determined as the heaviest weight that was successfully lifted. One repetition maximum testing was performed for chest press, seated row, shoulder press, leg press and leg extension. All 1 repetition maximum testing and training occurred in the same exercise laboratory.

### 4.4. Blood Analysis

For detailed information on the blood handling and assays, see our previously published study [25]. In brief, a certified phlebotomist drew approximately 10 mL of blood from the participants antecubital vein via venipuncture. The vacutainer tubes containing the whole blood and anticoagulant (ethylene diamine tetra-acetic acid, EDTA) were then inverted and centrifuged for 15 min at 1500× *g* at 4 °C to isolate plasma. The resulting plasma was aliquoted into microtubes that were stored in a −80 °C freezer until assays were performed. Multiplex technology was used to analyze apelin, FGF-21, IL-4, IL-6, IL-7, IL-15, LIF, and irisin. A Luminex MAGPIX flow cytometer (Luminex Corp., Toronto, ON, Canada) was used to measure IL-4, IL-6, and IL-7 (Human High Sensitivity T-Cell Magnetic Bead Panel assay; Milliplex Map Kit, EMD Millipore Corp., Billerica, MA, USA), LIF and irisin (Human Myokine Magnetic Bead Panel assay; Milliplex Map Kit, EMD Millipore Corp.), and apelin, IL-15, and FGF-21 (Human Myokine Magnetic Bead Panel assay; Milliplex Map Kit, EMD Millipore Corp.). All myokines were determined in duplicate.

### 4.5. Data Analysis

All data are presented as mean ± standard deviation unless otherwise noted. Initially, descriptive statistics were calculated. Subsequently, a Shapiro–Wilk test was performed to determine whether outcome measures were normally distributed, and a log-10 transformation was performed for those which were not normal. Outcome measures which were log transformed for statistical analysis are presented as absolute values in Table 1 to illustrate the absolute concentrations in circulation. Baseline anthropometric characteristics of the two groups were compared with an independent samples T-Test. Two-way repeated measure ANOVAs (age group × training status) were performed. If Mauchly’s Test of Sphericity was violated, a Greenhouse–Geisser correction was performed. If a significant interaction or main effect was identified, a Tukey’s post hoc test was used to determine the source of the difference. All statistical analyses were performed with Statistica (version 13.3, TIBCO Data Science/Statistica^TM^, Hamburg, Germany).

## Figures and Tables

**Table 1:** Resting myokine concentrations in younger and older males both pre- and post-12 weeks of resistance-exercise training.

Variable	Younger (n = 8)		Older (n = 7)		*p*-Values		
	Pre-Training	Post-Training	Pre-Training	Post-Training	Training Status	Age Group	Age Group × Training Status
Apelin (pg/mL)	476.0 ± 155.8	521.8 ± 141.9	363.5 ± 110.6	529.0 ± 135.9	**0.003**	0.432	0.066
Fibroblast Growth Factor—21 (pg/mL)	266.5 ± 148.6	262.5 ± 151.6	157.5 ± 61.6	183.6 ± 42.7	0.711	0.100	0.616
Interleukin-4 (pg/mL)	19.7 ± 17.1	20.1 ± 15.6	139.9 ± 190.4	118.2 ± 164.7	0.675	0.153	0.516
Interleukin-6 (pg/mL)	1.2 ± 1.6	1.7 ± 2.7	15.0 ± 21.9	13.6 ± 20.3	0.758	0.197	**0.041**
Interleukin-7 (pg/mL)	3.0 ± 1.6	3.4 ± 2.2	3.3 ± 1.4	3.4 ± 1.3	0.418	0.873	0.593
Interleukin-15 (pg/mL)	15.0 ± 4.9	17.6 ± 5.2	11.1 ± 2.6	17.5 ± 2.2	**<0.001**	0.299	0.066
Irisin (pg/mL)	447.7 ± 191.7	548.0 ± 222.4	363.2 ± 112.3	278.3 ± 127.1 *	0.817	0.053	**0.014**
Leukemia inhibitory factor (pg/mL)	23.4 ± 20.4	26.0 ± 9.4	17.5 ± 6.5	15.2 ± 4.5	0.395	0.221	0.076

* Indicates significantly lower than younger adults at the post-training time point. All significant *p*-values (*p* ≤ 0.05) are identified in bold.

## Data Availability

The data presented in this study are available upon request from the corresponding author.
